# Motivational and Ideological Underpinnings of Welfare Preferences in Eastern and Western Europe

**DOI:** 10.5964/ejop.v12i1.1045

**Published:** 2016-02-29

**Authors:** Márton Hadarics

**Affiliations:** a Political Psychology Research Lab, Eötvös Loránd University, Budapest, Hungary; University of Liverpool, Liverpool, United Kingdom

**Keywords:** motivation, selflessness, conventionality, ideology, welfare services, distributional justice, postsocialist economic system nostalgia

## Abstract

In our study we investigated the motivational and ideological correlates of the approval of welfare services in postsocialist Central Eastern and Western Europe. In the centre of our inquiry stood how the motivations of selflessness and conventionality, along with distributional justice principles, are related to our welfare preferences beyond our rational self-interest, furthermore, how these associations depend on social-cultural circumstances. We have found that the motivational background of egalitarian economic and welfare attitudes are substantially different in the two regions. While beside of the rationalisation of self-interest, it seems to be related to selflessness-driven solidarity in Western Europe, pro-welfare and egalitarian distributional views are primarily motivated by conventionality-driven norm adherence in postsocialist countries in the form of the mechanism of postsocialist economic system nostalgia. Our results highlight the benefits of a context-specific ’motivated social cognition’ approach to ideological and political attitudes.

## Introduction

One of the most important tasks of all democratic states is to ensure the emergence of two basic democratic values for their citizens, freedom and equality. Although these two values can contradict each other, just like in the case of *welfare services*. By these services the state tries to ensure not just equal rights and opportunities, but also the equality of life conditions, mainly in order to alleviate economic inequalities evolved due to free market competition (Thomassen, 2007). Substantial variance can be found among different societies regarding how much they emphasize the basic values of freedom and equality compared to each other, and these differences strongly influence welfare preferences in any given society. According to Fuchs and Klingemann (2002) the two extremities among Western democracies are represented by the „libertarian” United States and the young „socialist” Eastern European democracies in this regard, and the „liberal” Western European democracies are taking place in between. Since very different ideological elements pervade public thinking in these countries, it can be assumed that the psychologically relevant *motivational characteristics* underlying personal welfare preferences influence expectations towards the state through the acceptance or rejection of very different ideological elements, depending on social and cultural features of a specific country. Essential differences might be discovered between the younger Central Eastern European and the older Western European democracies in this regard.

Substantial individual differences can be found in welfare expectations towards the state, hence the question arises, what kind of motivational forces we can discover in the background as explanations for these deviations in public expectations. Former studies have identified a number of psychologically relevant motivational factors that are closely related to personal preferences regarding welfare services.

### Self-Interest

According to one of the most plausible explanations, a specific welfare service is supported mostly by those who have the opportunity to economically gain from it. This argument is built mainly on the *homo economicus* concept of rational choice theories, suggesting that the primary motivation for all of us is to maximize our own gains. In point of welfare services it means, those are much more in favour of a specific welfare service who share in it or feel themselves at risk of needing it at some point. That can explain why largescale governmental redistribution and a wide range of welfare services are supported much more by citizens with lower income, and by groups in an unfavourable position in the social hierarchy (Iversen & Soskice, 2001; Svallfors, 1997). Beside of this, it is a recurring finding that universal welfare services equally available for all citizens (e.g. education and healthcare services) partake of a significantly higher level of public support than means tested benefits with a limited scope of beneficiaries, like unemployment benefits (Blekesaune & Quadagno, 2003; Skocpol, 1991).

The direct relationship between the motivation of short-term self-interest and personal welfare preferences has been widely revealed and described in social sciences so far. At the same time, it has become obvious that the role of self-interest is not sufficient enough to explain the motivational background of individual differences in attitudes towards the welfare state utterly on its own, and it is necessary to take the role of other motivations into account.

### Selflessness, Empathy and Altruism

In social settings, one of the most important motivational factors that can prevent us from following our selfish interests is caring about the well-being of others. Is has been recognized in social justice research for a while now that those possessing power and resources are often willing to help the needy, even if it is out of accord with their own rational self-interest (see Blader & Tyler, 2002; Jost & Kay, 2010 for a review). In accordance with that, several studies showed that people with psychological characteristics making them to care about and emphatically identify with the difficult situation of others – people with a higher level of *selflessness motivation* – are much more in favour of governmental welfare services and redistribution (Duriez, Van Hiel, & Kossowska, 2005; Kulin & Svallfors, 2013; van Oorschot, 2002).

### Social Beliefs – Ideology, Social Justice and Stereotypes

Opinions about the necessity of governmental welfare services and benefits are in close connection with other ideological beliefs. One of the most important of these is the principle of allocation that is adhered by a respondent when making a judgment about the fairness of the distribution of resources within the society. Two distributional justice principles must be highlighted here, the principles of *equity* and *need*. According to the principle of equity, everyone should receive rewards in proportion to the invested resources, while this proportionality can be modified based on the principle of need, in order to help those in the most difficult situation (Deutsch, 1985). Corresponding with this pattern, it is a reccuring finding, that those relying on the equity principle oppose more of any kind of governmental provision and tend to tolerate economic-income inequalities, considering them as just. But those emphasizing the principle of need are much more in favour of largescale governmental distribution, income equality, equal availability of welfare services, and helping the most needy groups by means tested benefits (e.g. Kangas, 2003; Sabbagh & Vanhuysse, 2006; Sachweh, Ullrich, & Christoph, 2007).

Social beliefs and *stereotypes* about the recipient group of a specific welfare benefit can determine public support for that given provision too. This is a substantial factor in the case of selective means tested benefits, like unemployment benefits or income support. The basic question here is, whether the target group of a given benefit is perceived as deserving based on the social stereotype constructed about it. Several *deservingness criteria* have been identified that people consider while making a judgement about this question (De Swaan, 1988; van Oorschot, 2000). The criteria of *control* is especially important in this regard, which is an attributional decision about whether a disadvantaged group has gotten into an unfavourable position because of external circumstances or its own typical characteristics and acts. Welfare services that are targeting groups perceived as unable to significantly influence their own life circumstances (e.g. children, old, sick and disabled people), seem to partake in a higher level of public support compared to others, irrespective of social and cultural factors in a given society. At the same time, more citizens think that groups like the unemployed, poor, or immigrants are responsible for their own disadvantaged economic and social status, consequently, they do not deserve the help of the state. But those explaining poverty and unemployment with external factors, and not tracing them back to negative stereotypes about these groups (like to the lack of efforts or ambitions, or the „culture of poverty”), are more in favour of the governmental relief of these groups (e.g. Applebaum, 2001; Lepianka, van Oorschot, & Gelissen, 2009; van Oorschot, 2008).

It is important to emphasize that social beliefs like distributional justice principles, political and ideological preferences, stereotypes and autostereotypes are not independent from each other, but they are organized into a clear pattern (e.g. Hunyady, 2004; Jost, Glaser, Kruglanski, & Sulloway, 2003). Welfare preferences are important elements of this pattern. The study of Sabbagh and Vanhuysse (2006), based on the examination of eight countries, argues that attitudes towards welfare services are organized into two negatively interrelating *ideological metaframes*. Central elements of the first *„market-based”* metaframe are belief in individualism, meritocracy, work ethic and internal attribution of inequality, while the most significant elements of the second *„welfare-statist”* frame are egalitarian redistribution, a broad scope of welfare and external attribution of social inequality. Another major result of this study was that although structural relationships between the ideological elements were identical in the eight examined countries, but there was a great variance among these countries regarding how strongly their residents approved the two ideological metaframes. It means that the social, political, and cultural characteristics of a given country might influence welfare preferences of its citizens on their own, independently of any other factors.

### The Determining Role of Cultural Background – ’Socialist Legacy’?

A wide range of international comparison studies showed that although citizens of all societies are divided in their welfare preferences, however, these attitudes are explored on an aggregated level, systematic differences can be revealed between specific countries and groups of countries too. Residents of certain countries are much more in favour of largescale governmental redistribution and a wide scope of universally available welfare services than residents of other countries. Many authors trace this variance back to differences in the cultivated values and norms among these societies.

It is worthwhile to note that Western countries with old democratic traditions show a great variance regarding the welfare attitudes of their citizens (e.g. Arts & Gelissen, 2010; Blekesaune & Quadagno, 2003; Espig-Andersen, 1990; Sabbagh & Vanhuysse, 2006). At the same time the question arises, how have the historical experiences of the past, the *’socialist legacy’* affected welfare preferences of the citizens of the formerly socialist young democracies? In these countries the socialist state tried to eliminate economic-income inequalities by centralized dictatorial measures. This endeavor could be considered as a success in many regards, since in the years directly preceding the regime change, the Central Eastern European countries could be reckoned among the most equals in the world in terms of income distribution. If the assumption is true that the established economic and political institutional system has an effect on social beliefs and attitudes per se, then this must be observable in the public thinking of postsocialist countries too.

Two elements of the Central Eastern European public thinking seem to be especially peculiar. The common base of both elements is the substantial public need for governmental redistribution and paternalism. Several international comparative studies confirmed that residents of the formerly socialist countries endorse more *egalitarian* economic attitudes than their Western European peers, they prefer smaller income inequalities, and a stronger governmental role in the establishment and maintenance of that sort of equality. In addition, Eastern European citizens hold more exaggerated expectations towards the state to provide a wide range of *welfare services* and provisions as well. These differences between the Eastern and Western European public thinking had been observed in the years following directly after the regime change (Andreß & Heien, 2001; Austen, 2002; Blanchflower & Freeman, 1997; Corneo & Grüner, 2002; Delhey, 1999; Evans, 1998; Mason, 1995), and as it seems, have endured to this day, especially in the case of income egalitarianism (Alesina & Fuchs-Schündeln, 2007; Lipsmeyer & Nordstrom, 2003; Redmond, Schnepf, & Suhrcke, 2002; Roosma, Gelissen, & van Oorschot, 2013; Suhrcke, 2001).

### Conventionality and Postsocialist Economic System Nostalgia

The above mentioned differences in ideological preferences between Eastern and Western Europe can easily modify the effect of a particular psychological characteristic with a significant influence on personal welfare preferences, namely the motivational force of *conventionality*. Conventionality can be defined as a basic need to adhere to social conventions that are normative to accept in a given society. For someone with a high conventionality motivation, it is important to behave and act in accordance with normative views and conventions, which behaviour can fill one with the feeling of security and predictability.

Several psychological traits have been described so far, which make us more likely to conform ourselves to dominant social and ideological norms. Such traits are *conscientiousness, social conformity, (right-wing) authoritarianism, a low level of openness* (see Duckitt, 2001; Sibley & Duckitt, 2008), *dogmatism* (Rokeach, 1960), *need for structure* (Schaller, Boyd, Yohannes, & O’Brien, 1995), or *need for closure* (Kruglanski, 2004). According to the *system justification theory* (Jost & Banaji, 1994), and the theory of *ideologies as motivated social cognitions* (Jost et al., 2003) these psychological characteristics, by enhancing norm adherence, make us to accept and approve the social, political, and economic system we live in, even if it is contradictory to our economic self-interest (Jost & Hunyady, 2005). The original intention of the system justification theory was to provide a psychological explanation to the political behaviour of disadvantaged groups in the United States. According to the theory, by accepting ideological elements like *belief in meritocracy*, *the fairness of free market,* or *social mobility*, these groups contribute to the maintenance of the unequal social and economic system. Nevertheless the specific ideological elements to be accepted due to conventionality follow from dominant views in public thinking regarding a particular topic. That is why studies from formerly socialist countries – like Russia, Hungary, Serbia or Poland – showed that psychological traits related to conventionality correlate with the support for income egalitarianism, governmental redistribution, and economic intervention (see Duriez et al., 2005; Golec, 2002; Korzeniowski, 2006; Kossowska & Van Hiel 2003; McFarland, Ageyev, & Abalakina-Paap, 1992; Todosijević, 2008). Although these studies mostly ignored the role of rational self-interest, still they raise the possibility of a phenomenon that we can call *postsocialist economic system nostalgia*, a mechanism during which residents of a postsocialist country with a strong conventionality motivation are willing to approve income redistribution and welfare services, even if it is contradictory to one’s own self-interest.

### Our Study

The primary intention of our study presented hereunder was to reveal possible differences between Eastern and Western Europe on a whole regional level regarding how welfare preferences are influenced by the motivational factors of *self-interest, selflessness* and *conventionality*, and to determine through which social-ideological beliefs they affect welfare attitudes. We presumed that while economic-income egalitarianism is present in both regions, its motivational bases will differ due to the mechanism of postsocialist economic system nostalgia. This presumption was tested via the approval of two welfare services. One of these was the governmental provision of healthcare services, which is a universally available service, while the other was a selective means tested service, namely unemployment benefits.

## Method

### Sample

Our research was based on the 2008/09 survey database of the *European Social Survey (ESS)* programme. The European Social Survey is an academically driven iternational survey programme that has been conducted every two years with the participation of more than 30 European countries since 2001. The survey measures the attitudes, beliefs and behaviour patterns of diverse populations across a wide range of European countries, which makes it an ideal tool for international comparative studies. On the basis of the 2008/09 database we created an Eastern European (*N* = 11367) and a Western European sample (*N* = 11546), each including data of the representative samples of 6-6 countries. The Eastern European sample included data from Bulgaria, the Czech Republic, Poland, Hungary, Romania and Slovakia, while the Western European sample from Belgium, France, the Netherlands, Ireland, the United Kingdom and Switzerland.

### Measures

All variables were taken directly or calculated from the questionnaire of the fourth (2008/09) round of the ESS research programme. A number of ESS items have an inverse scoring, in case of such items the scoring was reversed so that the higher scores mark the greater extent of agreement with the content of the given item.

#### Approval of Welfare Services

In the case of both services, respondents had to sign their level of approval of these services on an 11-point scale. They were asked, how much responsibility they think governments should have to *“ensure adequate health care for the sick”* and *“ensure a reasonable standard of living for the unemployed”*, where “0” indicated *“Should not be governments' responsibility at all”* and “10” indicated *“Should be entirely governments' responsibility”*.

#### Self-Interest

The role of rational self-interest was caught by creating a dichotomous dummy variable for both welfare services. Respondents were categorized as concerned in their self-interest relating to unemployment, if they had reported at least one of the following conditions:

- is currently unemployed,
- has already been unemployed and seeking work for a period of more than three months,
- it is likely that during the next 12 months he/she will be unemployed and looking for work for at least four consecutive weeks,
- his/her partner is currently unemployed,
- main source of income in household is unemployment benefit.

Someone was categorized as concerned in one’s self-interest relating to healthcare, if one had reported at least one of the following conditions:

- described his/her health in general as “bad” or “very bad”,
- is hampered in his/her daily activities in any way by any longstanding illness, or disability, infirmity or mental health problem “a lot” or “to some extent”,
- is permanently sick or disabled,
- his/her partner is permanently sick or disabled.

#### Selflessness Motivation

To measure selflessness, an index was created based on three items of the *Human Values Scale* applied in the ESS core questionnaire. These items assessed how important is the well being of others for the respondents, how opened they are to the perspective and needs of others. Respondents were asked to indicate on a 6-point scale how much they agreed with the following statements:

- “She/he thinks it is important that every person in the world should be treated equally. She/he believes everyone should have equal opportunities in life.”
- “It is important to her/him to listen to people who are different from her/him. Even when she/he disagrees with them, she/he still wants to understand them.”
- “It's very important to her/him to help the people around her/him. She/he wants to care for their well-being.”

#### Conventionality Motivation

To measure this motivation, an index was created based on five items of the *Human Values Scale*. The items revealed how important it is to follow normative views and conventions, which can fill respondents with the feeling of security and predictability. Respondents indicated on a 6-point scale how much they agreed with the following items:

- “She/he believes that people should do what they're told. She/he thinks people should follow rules at all times, even when no-one is watching.”
- “It is important to her/him to be humble and modest. She/he tries not to draw attention to herself/himself.”
- “It is important to her/him always to behave properly. She/he wants to avoid doing anything people would say is wrong.”
- “It is important to her/him to live in secure surroundings. She/he avoids anything that might endanger her/his safety.”
- *“Tradition is important to her/him. She/he tries to follow the customs handed down by her/his religion or her/his family.”* (Cronbach-α values for the motivational indices are presented in Table 1)

#### Approval of the Distributional Principles of Meritocracy and Income Egalitarianism

Approval of meritocracy was measured by the following item: *“Large differences in people's incomes are acceptable to properly reward differences in talents and efforts.”*, while the following statement indicated the acceptance of income equality: *“For a society to be fair, differences in people's standard of living should be small.”* Respondents signed the level of acceptance on a 5-point scale.

#### Deservingness Criteria – Attributional Explanation for Unemployment

At the examination of the approval of unemployment benefits, social perception of the benecifiary group was considered to be important. We tried to assess the deservingness criteria of control through the following item, which was answered on a 5-point scale: *“Most unemployed people do not really try to find a job.”*

#### Demographics

In our statistical modeling procedure the effects of *gender*, *age*, *educational level, subjective and objective income levels* were also taken into account. Since the education systems of the countries making up the sample differ to a great extent, we defined education through the number of years spent with studies. In the case of objective income situation the individual indicated on a scale his/her position on the basis of the whole income of his/her household. This 10-degree scale was developed individually for each country, following the same methodology. Each degree of the scale means one decile of income distribution on the basis of the median income as a point of reference.^i^ In the case of subjective income respondents were asked how they feel about their household's income. They could select one of four levels from the followings:

- “Living comfortably on present income.”
- “Coping on present income.”
- “Finding it difficult on present income.”
- “Finding it very difficult on present income.”

**Table 1:** Descriptive Statistics of Samples

Variable	Eastern Europe			Western Europe		
	*M/N*	*SD*	α	*M/N*	*SD*	α
Gender						
Men (*N*)	5086	-	-	5331	-	-
Women (*N*)	6281	-	-	6215	-	-
**Age**	48.07	18.059	-	48.36	18.379	-
**Years of education**	11.90	3.468	-	12.91	3.992	-
**Objective income**	4.92	2.310	-	5.75	2.700	-
**Subjective income**	2.54	.855	-	1.81	.785	-
**Conventionality**	4.50	.792	.703	4.18	.866	.675
**Selflessness**	4.59	.791	.602	4.88	.717	.611
**Support for unemployment benefits**	6.77	2.581	-	6.24	1.987	-
**Support for healthcare services**	8.59	1.989	-	8.23	1.640	-
**Meritocracy**	3.41	1.120	-	3.38	1.043	-
**Egalitarianism**	3.77	.981	-	3.44	.976	-
**Stereotype - Unemployed**	3.29	1.133	-	3.04	1.066	-
Self-Interest						
Unemployment benefits (*N*)	4523	-	-	3783	-	-
Healthcare services (*N*)	3242	-	-	2871	-	-

### Procedure

In order to investigate the associations we were interested in, we applied structural equation modelling (SEM) procedure that is based on the analysis of covariances between variables (see Table 2). Separate so called saturated pathway models were set to explain the public approval of unemployment benefits and healthcare services both for the Eastern and the Western European samples, consequently, four models were created. Motivational and demographic variables were set as input variables in all of the models, and were assumed to influence attitudes towards welfare services directly, and indirectly via distributional preferences and stereotypes towards the unemployed.

**Table 2:** Covariances Between Variables

Variable	1.	2.	3.	4.	5.	6.	7.	8.	9.	10.	11.	12.	13.	14.
**1. Conventionality**	*E: .629* *W: .750*	.378*** (.007)	.263*** (.015)	.265*** (.019)	.036*** (.003)	-.020*** (.004)	.019* (.008)	.159*** (.007)	-.024** (.008)	.056*** (.004)	3.50*** (.138)	-.155*** (.026)	-.116*** (.017)	.050*** (.006)
**2. Selflessness**	.201*** (.006)	*E: .627* *W: .514*	.235*** (,015)	.154*** (.019)	-.001 (.003)	-.005 (.004)	.014 (.008)	.092*** (.007)	-.010 (.008)	.044*** (.004)	.647*** (.134)	.285*** (.026)	.134*** (.017)	-.020*** (.006)
**3. Support for healthcare services**	.030* (.013)	.118*** (.011)	*E: 3.955* *W: 2.690*	1.840*** (.051)	.063*** (.008)	.022* (.009)	-.094*** (.021)	.317*** (.019)	-.145*** (.021)	.036*** (.009)	2.397*** (.338)	-.504*** (.064)	-.306*** (.043)	.159*** (.016)
**4. Support for unemployment benefits**	.052*** (.016)	.157*** (.013)	1.070*** (.032)	*E: 6.662* *W: 3.948*	.063*** (.011)	.077*** (.012)	-.632*** (.028)	.616*** (.024)	-.147*** (.027)	.047*** (.012)	1.743*** (.438)	-1.545*** (.085)	-.656*** (.056)	.467*** (.021)
**5. Self-Interest – Healthcare services**	.030*** (.003)	.006* (.003)	.029*** (.007)	.040*** (.008)	*E: .204* *W: .187*	-.021*** (.002)	-.010* (.005)	.039*** (.004)	-.036*** (.005)	.015*** (.002)	3.363*** (.083)	-.340*** (.015)	-.282*** (.010)	.077*** (.004)
**6. Self-Interest – Unemployment benefits**	-.034*** (.004)	-.003 (.003)	.024*** (.007)	.057*** (.009)	.005* (.002)	*E: .240* *W: .220*	-.040*** (.005)	.012** (.005)	-.023*** (.005)	-.007** (.002)	-2.135*** (.085)	.003 (.016)	-.100*** (.011)	.067*** (.004)
**7. Stereotype - Unemployed**	.146*** (.009)	-.038*** (.007)	-.130*** (.016)	-.522*** (.020)	.025*** (.004)	-.026*** (.005)	*E: 1.283* *W: 1.136*	-.075*** (.010)	.093*** (.012)	-.002 (.005)	-.033 (.192)	.349*** (.037)	.379*** (.025)	-.175*** (.009)
**8. Egalitarianism**	.078*** (.008)	.101*** (.007)	.124*** (.015)	.311*** (.018)	.023*** (.004)	.006 (.004)	-.022* (.010)	*E: .963* *W: .952*	-.210*** (.010)	.026*** (.005)	2.216*** (.168)	-.684*** (.033)	-.322*** (.021)	.186*** (.008)
**9. Meritocracy**	.070*** (.008)	-.044*** (.007)	-.087*** (.016)	-.275*** (.019)	-.026*** (.004)	-.027*** (.005)	.069*** (.010)	-.257*** (.010)	*E: 1.253* *W: 1.087*	-.012* (.005)	-1.690*** (.190)	.282*** (.037)	.102*** (.024)	-.142*** (.009)
**10. Gender**	.019*** (.004)	.051*** (.003)	.000 (.008)	.010 (.009)	.004* (.002)	-.010*** (.002)	.015** (.005)	.028*** (.005)	-.033*** (.005)	*E: .247* *W: .248*	.572*** (.084)	-.081*** (.016)	-.108*** (.011)	.031*** (.004)
**11. Age**	4.13*** (.153)	.410*** (.123)	-.281 (.281)	1.310*** (.340)	2.101*** (.076)	-1.797*** (.082)	.886*** (.183)	1.423*** (.167)	-.358* (.178)	.138 (.085)	*E: 326.11* *W: 337.7*	-13.70*** (.601)	-12.43*** (.408)	2.58*** (.147)
**12. Years of education**	-.571*** (.033)	.082** (.027)	.417*** (.061)	.011 (.074)	-.249*** (.016)	.082*** (.017)	-.847*** (.040)	-.434*** (.036)	.171*** (.039)	-.048** (.019)	-16.76*** (.70)	*E: 12.02* *W: 15.93*	4.02*** (.084)	-1.05*** (.030)
**13. Objective income**	-.360*** (.022)	-.110*** (.018)	-.150*** (.041)	-.571*** (.050)	-.225*** (.011)	-.163*** (.012)	-.224*** (.027)	-.358*** (.025)	.218*** (.026)	-.167*** (.013)	-10.04*** (.471)	3.56*** (.106)	*E: 5.337* *W: 7.289*	-1.10*** (.021)
**14. Subjective income**	.046*** (.006)	.018*** (.005)	.082*** (.012)	.097*** (.015)	.050*** (.003)	.103*** (.004)	.058*** (.008)	.079*** (.007)	-.088*** (.008)	.018*** (.004)	-.749*** (.134)	-.482*** (.029)	-.991*** (.022)	*E: .730* *W: .615*

*Note.* Covariances and standard errors (in parantheses) in the Eastern European (E) sample are reported above the diagonal, covariances and standard errors in the Western European (W) sample are reported below the diagonal, variances are reported in italics along the diagonal.

*p < .05. **p < .01. ***p < .001.

The main reason for creating saturated models instead of unsaturated ones is that our primary inquiry was not about whether particular associations between the investigated motivations and ideological elements are observable in the two samples, we were much more curious about the relative strengths of these association between the two samples. We did not assume that a motivational variable might be related to an ideological element in one of the samples and not in the other, because all the investigated distributional and welfare beliefs function as integral elements of the public thinking in both regions. What we assumed is that particular associations might be stronger in one of the samples. To test this assumption, our aim was to verify the equivalence of certain effect sizes in the models between the two samples. For this aim, in each case a common two-grouped structural equation model was created based on the method described by Byrne (2010), wherein the strength of the relevant relationships between the two groups was fixed one by one on a permanent level. If the χ2–based fit index of the common model deteriorated to a significant extent with this restriction, it indicated that the examined effect strength differed between the Eastern and the Western European groups to a significant extent.

## Results

Before we examine the main findings of our SEM models, it is worth having a look at Table 3, where the national mean values are presented for the distributional justice principles and the support for welfare services. These values confirm the stronger egalitarian and paternalist preferences in the postsocialist public thinking compared to the Western European. Both at the support for healthcare services and unemployment benefits, mean values of the Eastern European countries are in the upper half of the rank order created from the national mean values, with the exception of Romania at the approval of healthcare services and Slovakia at unemployment benefits. The pattern is very similar at income egalitarianism – with the Czech Republic as an exception – while in the case of meritocracy there is a tendency for polarisation among postsocialist countries. It is worth noting in reference to the later result, that meritocracy was measured from a prescriptive view, not from a descriptive one. Respondents were asked, how important the principle of merit is to operate in the social sphere, and not to what extent it is operating currently in their country. Citizens of the postsocialist countries are much more sceptical in the later descriptive view (Csepeli, Örkény, Székelyi, & Barna, 2004; Örkény & Székelyi, 2000).

**Table 3:** National Mean Scores of Support for Welfare Services and Distributional Justice Principles

Egalitarianism	Meritocracy	Healthcare services	Unemployment benefits
Bulgaria: 3.96^a^	Romania: 3.85^a^	Bulgaria: 9.09^a^	Romania: 7.71^a^
Hungary: 3.94^a^	Czech Republic: 3.85^a^	Hungary: 9.03^a^	Bulgaria: 7.24^a^
Romania: 3.86^a^	Poland: 3.62^a^	Poland: 8.91^a^	Hungary: 6.85^a^
Slovakia: 3.77^a^	United Kingdom: 3.51	United Kingdom: 8.74	Ireland: 6.77
Switzerland: 3.62	Ireland: 3.44	Ireland: 8.58	Poland: 6.37^a^
Poland: 3.60^a^	Netherlands: 3.41	Slovakia: 8.39^a^	Czech Republic: 6.30^a^
Belgium: 3.53	Belgium: 3.37	Czech Republic: 8.38^a^	Netherlands: 6.30
France: 3.52	Switzerland: 3.35	Netherlands: 8.24	Switzerland: 6.27
Ireland: 3.47	Bulgaria: 3.34^a^	Belgium: 8.03	France: 6.13
Czech Republic: 3.45^a^	France: 3.21	France: 8.02	Belgium: 6.06
United Kingdom: 3.28	Slovakia: 3.03^a^	Romania: 7.88^a^	United Kingdom: 6.00
Netherlands: 3.23	Hungary: 2.56^a^	Switzerland: 7.66	Slovakia: 5.85^a^

*Note.* Mean scores are presented in a descending rank order.

^a^Eastern European country.

**Figure 1:**
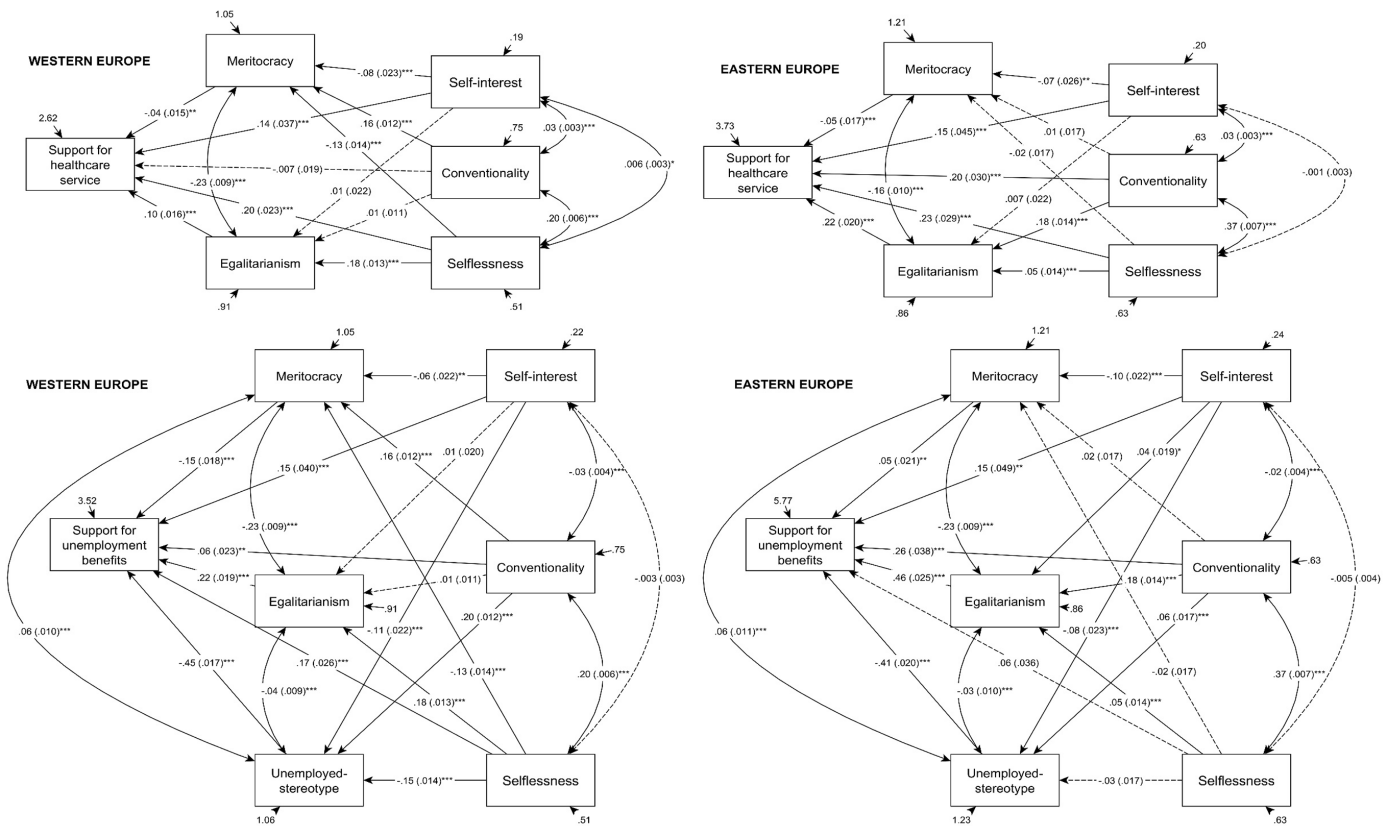
Saturated pathway models explaining support for welfare services in Western and Eastern Europe. *Note.* Unstandardized regression weights are followed by standard errors in parentheses. Demographic variables and their effects are not displayed for a better transparency of the charts. Own calculations based on ESS data. *p < .05. **p < .01. ***p < .001.

In the course of statistical modeling, so called saturated SEM models were created with all possible connections set between all variables (see Figure 1). In the model explaining approval of governmental healthcare services in the Eastern European sample, selflessness showed a positive relationship with both egalitarianism (*b_E-Eu_* = .05; *p* < .001) and the support for this welfare service (*b_E-Eu_* = .23; *p* < .001), but was not related to the acceptance of meritocracy (*b_E-Eu_* = -.02; *p* = .156). Conventionality related also positively to egalitarianism (*b_E-Eu_* = .18; *p* < .001) and the approval of healthcare services (*b_E-Eu_* = .20; *p* < .001), but showed no relationship with meritocracy (*b_E-Eu_* = .01; *p* = .287).

Beside of this, it is worth noting that both the acceptance of income egalitarianism and meritocracy had an effect on the approval of healthcare services, but in accordance with our expectations, with an inverse direction. In the postsocialist sample egalitarianism showed a positive relationship with the approval of this welfare service (*b_E-Eu_* = .22; *p* < .001), while meritocracy had a negative, and weaker effect on it (*b_E-Eu_* = -.05; *p* < .001).

In the model explaining support for healthcare services in the Western European sample, we could observe that selflessness related negatively to the acceptance of meritocracy (*b_W-Eu_* = -.13; *p* < .001), but enhanced income egalitarianism (*b_W-Eu_* = .18; *p* < .001) and the approval of the mentioned welfare service itself (*b_W-Eu_* = .20; *p* < .001). Conventionality related exclusively to the acceptance of meritocracy to a statistically significant extent (*b_W-Eu_* = .16; *p* < .001).

Just like in the Eastern European group, agreeing with the principle of meritocracy had a negative effect on the approval of healthcare services (*b_W-Eu_* = -.04; *p* = .007), and egalitarianism had a positive effect on the same variable (*b_W-Eu_* = .10; *p* < .001), but this later effect was significantly stronger in the Eastern European sample than in the Western European one (*Δχ^2^* = 23.4; *Δdf* = 1; *p* < .001).

We can see the regression coefficients from the models based on the national samples in Table 4. On the whole, these confirm our findings on the regional level. Conventionality enhanced the approval of income egalitarianism in all the Eastern European countries, while this connection was non-significant in every Western European national sample. On the other hand, the very same motivation intensified the acceptance of meritocracy in the Western European countries – except for the Netherlands – while there is a lack of this relationship in the Eastern European samples, apart from the Czech Republic.

**Table 4:** Regression Coefficients of Pathway Models Explaining Support for Healthcare Services Based on National Samples

Country	Egalitarianism			Meritocracy			Healthcare	
	Healthcare	Conventionality	Selflessness	Healthcare	Conventionality	Selflessness	Conventionality	Selflessness
Western Europe								
Belgium	.131*** (.037)	.027 (.036)	.168*** (.038)	-.035 (.034)	.183*** (.039)	-.131*** (.041)	-.028 (.054)	.332*** (.057)
United Kingdom	.140*** (.033)	.042 (.024)	.126*** (.028)	-.056 (.032)	.134*** (.024)	-.077** (.028)	.007 (.037)	.171*** (.043)
France	.135*** (.034)	-.012 (.030)	.158*** (.032)	-.087** (.031)	.251*** (.033)	-.182*** (.036)	-.063 (.046)	.329*** (.049)
Netherlands	.054 (.037)	.042 (.028)	.163*** (.033)	.036 (.035)	.050 (.030)	-.141*** (.035)	-.009 (.043)	.239*** (.050)
Ireland	.090** (.042)	.056 (.028)	.160*** (.034)	-.118** (.039)	.142*** (.031)	-.149*** (.036)	-.090 (.050)	.220*** (.059)
Switzerland	.309*** (.056)	-.014 (.024)	.208*** (.032)	-.045 (.047)	.129*** (.028)	-.078* (.038)	-.165** (.057)	.159* (.076)
Eastern Europe								
Bulgaria	.141*** (.039)	.220*** (.029)	.063* (.029)	.013 (.031)	.029 (.036)	-.024 (.035)	.164** (.053)	.178*** (.051)
Czech Republic	.140** (.044)	.230*** (.036)	-.006 (.035)	.203*** (.048)	.103** (.034)	.044 (.033)	.388*** (.073)	.193** (.069)
Poland	.261*** (.045)	.168*** (.034)	.027 (.036)	-.043 (.041)	-.019 (.037)	-.001 (.039)	.176** (.062)	.047 (.065)
Hungary	.204*** (.041)	.082* (.040)	.148*** (.037)	-.119*** (.036)	.032 (.046)	-.049 (.044)	.090 (.065)	.103 (.061)
Romania	.408*** (.059)	.196*** (.034)	.019 (.033)	.200*** (.061)	-.035 (.033)	.095** (.032)	.159 (.093)	.240** (.089)
Slovakia	.184*** (.053)	.108** (.038)	.098** (.037)	-.066 (.045)	-.047 (.045)	.043 (.037)	.439*** (.084)	.188* (.081)

*Note.* This table shows the strength of the relationships with standard errors between the distributive justice principles, the motivational variables and the support for healthcare services. Unstandardized regression weights are followed by standard errors in parentheses.

*p < .05. **p < .01. ***p < .001.

It is also interesting that conventionality had a direct positive effect on the approval of healthcare services in the postsocialist samples – although this effect was not significant in the case of Hungary and Romania – while this motivational variable influenced welfare preferences only in the case of Switzerland among the Western European samples, but here the direction of the relationship was the opposite, norm-follower respondents tended to disapprove healthcare services provided by the state.

We can also observe that from the two distributional principles income egalitarianism influenced attitudes towards healthcare services to a substantially greater extent in both regions than meritocracy did. The relationship between egalitarianism and pro-welfare opinions regarding healthcare services was absent exclusively in the case of the Netherlands, while meritocracy influenced these opinions to a significant extent only in the half of the Eastern European and in the third of the Western European samples.

After examining the public support for governmental healthcare services, if we have a look at the SEM models explaining unemployment benefits, we can find the followings in the case of Eastern Europe: selflessness was related neither to the attributional stereotype about the unemployed (*b_E-Eu_* = .03; *p* = .053), nor to meritocracy (*b_E-Eu_* = -.02; *p* = .172), and it showed no effect on the approval of unemployment benefits (*b_E-Eu_* = .06; *p* = .069). Contrarily, income egalitarianism was intensified by selflessness (*b_E-Eu_* = .05; *p* < .001), just like in the case of the model explaining support for healthcare services.

In this group, conventionality enhanced the approval of income egalitarianism (*b_E-Eu_* = .18; *p* < .001), an internal attributional explanation for unemployment (*b_E-Eu_* = .06; *p* < .001), and also the support for unemployment benefits (*b_E-Eu_* = .26; *p* < .001). At the same time, this motivational factor had no effect on the acceptance of meritocracy (*b_E-Eu_* = .02; *p* = .283).

If we examine how distributional justice principles influence the support for unemployment benefits, the following pattern can be revealed: unfavourable views about the unemployed moderated (*b_E-Eu_* = -.41; *p* < .001), while egalitarianism enhanced (*b_E-Eu_* = .46; *p* < .001) the approval of unemployment benefits. The variable of meritocracy stood apart from this pattern due to its moderate positive relationship to the approval of this selective benefit (*b_E-Eu_* = .05; *p* = .008).

In our saturated models explaining personal support for unemployment benefits in the Western European sample selflessness related positively to egalitarianism (*b_W-Eu_* = .18; *p* < .001), and this connection was significantly stronger in this group than in the Eastern European one (*Δχ^2^* = 42.3; *Δdf* = 1; *p* < .001). Selflessness also amplified the support for unemployment benefits (*b_W-Eu_* = .18; *p* < .001) but extenuated meritocratic beliefs (*b_W-Eu_* = -.13; *p* < .001) and malevolent stereotypes towards the unemployed (*b_W-Eu_* = -.15; *p* < .001). The motivation of conventionality showed a positive relationship to the acceptance of meritocracy (*b_W-Eu_* = .16; *p* < .001), to the approval of this particular welfare service (*b_W-Eu_* = .07; *p* = .003), and to the internal attributional stereotypes (*b_W-Eu_* = .20; *p* < .001). This later connection was identified also in the postsocialist group, but with a significantly weaker effect size (*Δχ^2^* = 47.5; *Δdf* = 1; *p* < .001). The direction of the connection between conventionality and the personal support for unemployment benefits was the same in both samples, but turned out to be stronger in the Eastern European region (*Δχ^2^* = 19.1; *Δdf* = 1; *p* < .001).

Beside of the results mentioned above, it is worth underlining that in the Western European sample meritocratic views (*b_W-Eu_* = -.15; *p* < .001) and unfavourable stereotypes attenuated (*b_W-Eu_* = -.45; *p* < .001), while income egalitarianism enhanced the approval of unemployment benefits (*b_W-Eu_* = .22; *p* < .001). The later two relationships were observable also in the postsocialist group, but income egalitarianism had a significantly stronger effect there (*Δχ^2^* = 58.8; *Δdf* = 1; *p* < .001), while there was no difference in the effect of stereotyping (*Δχ^2^* = 2.8; *Δdf* = 1; *p* = .092).

Our SEM models set up to the national samples highly confirm our findings on the regional level. Relevant regression coefficients from these models are presented in Table 5. Conventionality enhanced income egalitarianism in all the postsocialist countries again, while this connection was absent from the Western European countries. On the other hand, this motivation, just like in the case of healthcare services, intensified the approval of meritocracy in every Western European country – except for the Netherlands – while this relationship was missing in the Eastern European samples – except for the Czech Republic. Furthermore, conventionality showed a direct positive relationship with the investigated welfare service in the postsocialist samples – except for Hungary – while the very same connection was non-significant in the Western European models.

**Table 5:** Regression Coefficients of Pathway Models Explaining Support for Unemployment Benefits Based on National Samples

Country	Egalitarianism			Meritocracy			Unemployment	
	Unemployment	Conventionality	Selflessness	Unemployment	Conventionality	Selflessness	Conventionality	Selflessness
Western Europe								
Belgium	.263*** (.047)	.029 (.036)	.164*** (.038)	-.109* (.043)	.184*** (.039)	-.126** (.041)	-.005 (.069)	.240*** (.073)
United Kingdom	.261*** (.046)	.043 (.024)	.125*** (.028)	-.237*** (.045)	.135*** (.024)	-.081** (.028)	.094 (.053)	.193*** (.060)
France	.270*** (.039)	-.014 (.030)	.158*** (.032)	-.137** (.035)	.251*** (.033)	-.183*** (.036)	-.032 (.053)	.174** (.056)
Netherlands	.141** (.045)	.042 (.028)	.164*** (.033)	-.056 (.043)	.049 (.030)	-.141*** (.035)	.039 (.052)	.186** (.061)
Ireland	.152** (.051)	.055 (.028)	.160*** (.034)	-.137** (.048)	.139*** (.031)	-.147*** (.036)	-.108 (.061)	.207** (.079)
Switzerland	.238*** (.054)	-.013 (.024)	.208*** (.032)	-.185*** (.046)	.126*** (.028)	-.079* (.038)	.013 (.056)	.115 (.074)
**Eastern Europe**								
Bulgaria	.385*** (.062)	.223*** (.029)	.058* (.029)	-.006 (.051)	.027 (.036)	-.018 (.035)	.189* (.084)	.257** (.082)
Czech Republic	.536*** (.050)	.230*** (.036)	-.005 (.035)	-.027 (.054)	.101** (.034)	.039 (.033)	.278*** (.082)	.052 (.078)
Poland	.460*** (.070)	.169*** (.034)	.024 (.036)	-.110 (.064)	-.017 (.037)	-.001 (039)	.298** (.096)	.084 (.100)
Hungary	.228*** (.062)	.082* (.040)	.148*** (.037)	.017 (.054)	.034 (.046)	-.048 (.044)	.139 (.098)	.176 (.092)
Romania	.385*** (.054)	.197*** (.034)	.022 (.033)	.081 (.056)	-.036 (.033)	.094** (.032)	.368*** (.086)	.185* (.083)
Slovakia	.264*** (.066)	.109** (.038)	.104** (.036)	.069 (.057)	-.048 (.045)	.023 (.042)	.391*** (.106)	-.186* (.101)

*Note.* This table shows the strength of the relationships and standard errors between the distributive justice principles, the motivational variables and the support for unemployment benefits. Unstandardized regression weights are followed by standard errors in parentheses.

*p < .05. **p < .01. ***p < .001.

Our data showed that selflessness has a much more obvious effect both on the distributional principles and the approval of unemployment benefits in the Western European region compared to the Eastern European one. This motivational variable showed a significant connection with both distributional principles in every Western European sample, and with one exception it was related to the support for unemployment benefits as well. On the other hand, selflessness was related to meritocracy only in one case among the Eastern European countries, and only in three-three cases to egalitarianism and pro-welfare views.

Regarding the distributional justice principles it is worth highlighting, that income egalitarianism fostered the personal support for unemployment benefits in every single investigated country, while meritocracy had the same effect in none of the Eastern European countries, but almost in all the Western European ones.

## Discussion

Based on our findings from the analysed database it seems that income egalitarianism and paternalism, which had been important elements of both the ideology and the practical fruition of socialism, still maintain a stronger normative force in the public thinking of the Central Eastern European postsocialist countries than in their Western European counterparts, more than twenty years after the regime change. The role of dissatisfaction with the new social, economic, and political system is incontestable in this phenomenon, since the disadvantageous changes in everyday life, the rapid lapse of security and predictability has fostered the nostalgic rehabilitation of economic egalitarianism and paternalism in the public thinking of this group of countries.

The two investigated distributional justice principles have unequivocally different embeddedness in the public thinking of the two regions. It is conspicuous that meritocracy is not related to the motivational variables or welfare preferences among the postsocialist countries, while the same distributional principle has an obvious relationship with the investigated motivations and the personal approval of unemployment benefits among the Western European countries. It seems that meritocracy has a deep embeddedness in the Western European public thinking, while this embeddedness seems to be absent from the Eastern European region. Since there is no considerable tradition of the ideological element of meritocracy in the postsocialist region, because it had not been the part of the social discourse during the socialist era, meritocracy could not became a significant element of motivated social and political cognition. Whatever individual motivations work in someone, those motivations do not stimulate the acceptance or refusal of meritocracy, and meritocracy does not substantially influence ideological views about the welfare state.

We have seen a stronger diversity among young postsocialist democracies in the overall level of the acceptance of meritocracy compared to the older European democracies. The more or less persistent and steady social and economic development in Western Europe could have created a more suitable environment for individual merit, talent, and effort to prevail in the perception of the society, while the rapid and drastic changes during and after the Eastern European regime change have not made it realistic to attribute the rise or fall of particular social groups to individual merit. Presumably that is why these countries differ from each other to such a great extent in the prescriptive approval of meritocracy, and that is why the principle itself lacks strong embeddedness in public thinking.

On the contrary, in Western Europe, with a considerably longer tradition of the idea of meritocracy, this ideological element constitutes an important part of motivated political cognition and reasoning. Both the motivations of selflessness and conventionality force people to choose a standpoint regarding the principle of meritocracy. It is interesting that in this region, from the two welfare services, the approval of unemployment benefits was influenced by the personal acceptance of meritocracy to a larger extent than the other welfare service. In case of a selective welfare service it might become a significant question, whether the target group deserves public help. That is why those generally preferring a merit-based distribution can reject a selective means tested policy. On the contrary, universal welfare services are not related to intergroup conflicts, hence opinions about these services are less likely to be influenced by ideological elements that are conserving and legitimizing the existing group hierarchy, just like meritocracy.

Income egalitarianism has shown a positive relationship to the support for both welfare services in both regions. This is understandable in the case of healthcare services, since this welfare benefit is equally available for all citizens because of its universality. That is why someone preferring the distributional principle of equality, will presumably support this sort of welfare services. Beside of this, in Western European countries with a long tradition of free market, income egalitarianism can function as a counterweight for other ideological elements that are legitimizing the existing social status quo. That is how income egalitarianism can affect attitudes towards selective welfare services like unemployment benefits, where social group hierarchy can be a highly relevant question. As *social dominance theory* suggests, in this case ideological elements like meritocracy or unfavourable stereotypes about disadvantaged groups can function as *hierarchy-enhancing legitimizing myths*, while income egalitarianism can be considered as a *hierarchy-attenuating myth* to balance the former ones (Sidanius & Pratto, 1999).

Naturally we can not rule out that income egalitarianism serves the function described above also in the postsocialist Central Eastern European societies. Although our results are indicating an alternative explanation, namely that in this region the aforementioned ideological element affects welfare preferences by the mechanism of *postsocialist economic system nostalgia*. This conclusion becomes obvious in the first place, if we review how the motivational variables of selflessness and conventionality affect the acceptance of ideological elements. This pattern is specifically suggestive in the case of conventionality, which was related to normative ideological contents possessing a significant embeddedness in the public thinking of the given region of Europe. This ideological content was meritocracy in Western Europe, and economic egalitarianism and governmental paternalism in Eastern Europe. It seems that in Western European countries conventionality stimulates people to accept the principle of meritocracy, and through that to oppose selective welfare services independently from the motivational force of economic self-interest. The phenomenon of conventionality-driven *economic system justification* can be identified in this pattern, as it is described by *system justification theory* (Jost & Hunyady, 2005). In the course of this, Western European citizens with a heightened conventionality motivation rationalize and conserve existing economic inequalities by approving legitimizing ideologies like meritocracy, independently from their rational self-interest, or even directly contradictory to that.

On the other hand, in the postsocialist countries the assumed mechanism of conventionality-driven *postsocialist economic system nostalgia* seems to show up. It is similar in the two mechanisms that in both cases the very same motivation stimulates people to act even against their rational economic interests by accepting social beliefs that provide the sense of certainty and predictability by their normative nature. The difference is that in long-established liberal democracies these normative economic beliefs are mostly related to free market capitalism, while in postsocialist countries these views are attached to the economic system of the past, not the present. In this sense, we can talk about a timely shifted economic system justification that is based on the egalitarian and paternalist ideas of socialism, which could preserve their normative force against the principles of the currently existing economic system due to the general dissatisfaction with the present.

We can see that income egalitarianism plays a significant role in the postsocialist public thinking, but to understand the mechanism of postsocialist economic system nostalgia, it is important to see its motivational background on the whole. In the Western European region selflessness consistently correlated positively with the acceptance of income egalitarianism, and pro-welfare attitudes, and negatively with meritocracy and disadvantageous stereotypes towards the unemployed. However it is not completely true in the young postsocialist democracies, since though selflessness was related to the approval of governmental healthcare services in the case of four countries, but the same motivation showed a positive connection to the support for unemployed benefits only in two national samples, and did not in the others. Moreover, selflessness was unrelated to the acceptance of meritocracy in the Eastern European region, and it enhanced the approval of income egalitarianism only in three national samples, what adds up to a substantially weaker relationship between these two variables in the Eastern European region compared to Western Europe.

This is an important result, because as we have seen, income egalitarianism is a significant predictor of welfare preferences and expectations towards the state in both regions, regardless of the selective or universal nature of welfare services. But we could reveal a widely different motivational background behind the expressed egalitarian attitudes. *This motivational background seems to be selflessness-based social solidarity in Western Europe, but conventionality-based norm adherence in Central Eastern European.* In the postsocialist region selflessness does not show a consistent relationship with egalitarian political-ideological beliefs like income egalitarianism or the rejection of meritocracy, or with tolerant opinions towards particular disadvantaged groups, like the unemployed.

Of course, we have to mention some considerable limitations of our study, mostly ensuing from the nature of the analysed dataset and the selected research methods. First of all, we have to emphasize that our study was a correlational one, consequently we can not be completely certain about the causal relationships between the investigated variables. For example, while it seems perfectly sensible to assume that our motivations and more abstract level value preferences influence our attitudes towards specific welfare policies, others assume also that the actual operation of the welfare institutional system can influence value preferences within any given society (Espig-Andersen, 1990). Another serious limitation of our study is the operationalisation of the investigated variables. While we have to stress that analysing ESS data is a perfect tool to maximise the external validity of our results, we also have to admit that construct validity is not the most significant strength of such a large-scale international survey programme. Most of the selected variables were measured only by one single item, and not by thoroughly constructed and carefully validated attitude and personality scales.

### Conclusions

Our findings seem to confirm that a more accurate description can be obtained about the psychological mechanism of attitude formation regarding the welfare state, if we consider these attitudes as results of a motivated social cognition process (see Jost et al., 2003). This framework has been applied to a surprisingly limited extent by political science and sociology so far, the motivational base of individual welfare preferences has been identified mostly in the form of rational economic self-interest. Nevertheless our results indicate that both selflessness and conventionality play a non-negligible role in our attitudes about welfare services, since they provide a convincing individual level psychological explanation to the acceptance or rejection of ideological element that are relevant to our expectations towards the welfare state.

As we could see, income egalitarianism is an important predictor for personal demands for welfare services in both investigated regions of Europe, but with a substantially different motivational background. This motivational background seems to be selflessness-based social solidarity in Western Europe, but individual motivations behind income egalitarianism are evidently more selfish in the Eastern European region, since primarily they consist of the rationalisation of personal economic self-interest and the mechanism of postsocialist economic system nostalgia. In the course of this, due to conventionality motivation, people conform to social beliefs that are normative in the public thinking of a given society. Income egalitarianism is such a belief, which is a deeply cultivated norm of the former system in postsocialist countries. In this system, there had been little chance for true social solidarity to evolve, because according to the social perception, it was the responsibility solely of the regime to ensure the well-being of the whole society, and social welfare was not perceived as a result of cooperation among different social groups. Consequently, the heightened economic egalitarianism and paternalism of postsocialist Central Eastern European countries can be regarded as resultants of the pursuit of rational self-interest and the mechanism of postsocialist economic system nostalgia, and not social solidarity. It is an interesting question, how the accumulating experiences in the new political and economic system will affect the motivational underpinnings of welfare attitudes, to what extent it will converge to the Western European pattern, or preserve its peculiarity.

Since providing appropriate and high-quality welfare services is one of the most significant and costly responsibilities of any national government, it is vital for decision-makers to have an extensive understanding of the deeper motivational context behind the particular views or opinions about welfare services and specific welfare policies. With this insight, political actors have the opportunity to enhance the quality of goal-oriented decision-making and to work out more precise welfare policy solutions that are in accordance with the specific expectations of their voters.

### Future Directions

As a future direction in our line of research, it would be important to investigate the specific causal relationships between individual motivations and cultivated ideological elements, including distributive preferences and welfare attitudes. By an experimental approach, it would be very interesting to test whether it is possible to enhance or reduce personal approval of specific welfare policies by the experimental manipulation of the individual-level motivations of selflessness and conventionality. Based on the results presented above, it is likely that if our aim is to modify or reinforce one’s opinion about a specific welfare policy, we have to do that by influencing different personal motivations, depending on the cultural and social context. In the Western European context we could probably reach this goal more efficiently by influencing selflessness, while in the postsocialist context manipulating the motivation of conventionality might be the appropriate way.

As an additional direction, it would be also fruitful to investigate the possible boundaries of social solidarity, and put it into a motivational framework. It can be assumed that people with different motivations perceive social boundaries regarding the entitlement for welfare services in a different way. As we saw, people with a higher level of conventionality motivation in Eastern Europe tend to approve governmental welfare services, because welfare paternalism is still an integral part of the postsocialist public thought. But we can assume that people with this motivation will be apt to exclude cultural and non-conventional outgroups from the scope of welfare entitlement, and preserve that only for those who can be regarded as reliable and conventional ingroup members. It is probable, because conventionality might make people to approve governmental paternalism and disapprove non-conventional outgroups at the same time in the postsocialist context. That is how conventionality can serve as a motivational base for welfare chauvinism in Eastern Europe, which might be a very important potential topic for future research.

## References

[r1] Alesina A. Fuchs-Schündeln N. (2007). Good bye Lenin (or not?): The effect of communism on people. American Economic Review, 97, 1507–1528. doi:. 10.1257/aer.97.4.1507

[r2] Andreß H.-J. Heien T. (2001). Four worlds of welfare state attitudes? A comparison of Germany, Norway, and the United States. European Sociological Review, 17(4), 337–356. doi:. 10.1093/esr/17.4.337

[r3] Applebaum L. D. (2001). The influence of perceived deservingness on policy decisions regarding aid to the poor. Political Psychology, 22(3), 419–442. doi:. 10.1111/0162-895X.00248

[r4] Arts, W. A., & Gelissen, J. (2010). Models of the welfare state. In F. G. Castles, S. Leibfried, J. Lewis, H. Obinger, & C. Pierson (Eds.), *The Oxford handbook of the welfare state* (pp. 568-583). New York, NY, USA: Oxford University Press.

[r5] Austen S. (2002). An international comparison of attitudes to inequality. International Journal of Social Economics, 29(3), 218–237. doi:. 10.1108/03068290210417106

[r6] Blader, S., & Tyler, T. R. (2002). Empathy and justice as reasons for helping victims. In M. Ross & D. T. Miller (Eds.), *The justice motive in everyday life* (pp. 226-250). Cambridge, United Kingdom: Cambridge University Press.

[r7] Blanchflower D. G. Freeman R. B. (1997). The attitudinal legacy of communist labor relations. Industrial & Labor Relations Review, 50(3), 438–459. doi:. 10.1177/001979399705000304

[r8] Blekesaune M. Quadagno J. (2003). Public attitudes toward welfare state policies: A comparative analysis of 24 nations. European Sociological Review, 19(5), 415–427. doi:. 10.1093/esr/19.5.415

[r9] Byrne, B. M. (2010). *Structural equation modelling with AMOS – Basic concepts, applications and programming* (2nd ed.). New York, NY, USA: Routledge.

[r10] Corneo G. Grüner H. P. (2002). Individual preferences for political redistribution. Journal of Public Economics, 83, 83–107. doi:. 10.1016/S0047-2727(00)00172-9

[r11] Csepeli, G., Örkény, A., Székelyi, M., & Barna, I. (2004). Blindness to success: Social psychological objectives along the way to a market economy in Eastern Europe. In J. Kornai, B. Rothstein, & S. Rose-Ackerman (Eds.), *Creating social trust: Problems of post-socialist transition* (pp. 213-240). New York, NY, USA: Palgrave Macmillan.

[r12] Delhey, J. (1999). *Inequality and attitudes: Postcommunism, Western capitalism and beyond* (Working Paper No. FS III 99-403). Berlin, Germany: WZB Berlin Social Science Center.

[r13] De Swaan, A. (1988). *In care of the state*. Amsterdam, The Netherlands: Bakker.

[r14] Deutsch, M. (1985). *Distributive justice: A social psychological perspective.* New Haven, CT, USA: Yale University Press.

[r15] Duckitt J. (2001). A dual-process cognitive-motivational theory of ideology and prejudice. Advances in Experimental Social Psychology, 33, 41–113. 10.1016/S0065-2601(01)80004-6

[r16] Duriez B. Van Hiel A. Kossowska M. (2005). Authoritarianism and social dominance in Western and Eastern Europe: The importance of the sociopolitical context and of political interest and involvement. Political Psychology, 26(2), 299–320. doi:. 10.1111/j.1467-9221.2005.00419.x

[r17] Espig-Andersen, G. (1990). *The three worlds of welfare capitalism*. Cambridge, United Kingdom: Polity Press.

[r18] European Social Survey. (2008). *ESS Round 4: European Social Survey Round 4 Data (2008)* (Data file edition 4.3). Bergen, Norway: Norwegian Social Science Data Services, Norway – Data Archive and distributor of ESS data.

[r19] Evans G. (1998). Britain and Europe: Separate worlds of welfare? Government and Opposition, 33, 183–198. doi:. 10.1111/j.1477-7053.1998.tb00789.x

[r20] Fuchs D. Klingemann H.-D. (2002). Eastward enlargement of the European Union and the identity of Europe. West European Politics, 25(2), 19–54. doi:. 10.1080/713869598

[r21] Golec A. (2002). Need for cognitive closure and political conservatism: Studies on the nature of the relationship. Polish Psychological Bulletin, 33(4), 5–12.

[r22] Hunyady, G. (2004). Social stereotypes and the "implicit social theory". In J. T. Jost, M. R. Banaji, & D. A. Rentice (Eds.), *Perspectivism in social psychology: The Yin and Yang of scientific progress* (pp. 187-201). Washington, DC, United States: American Psychological Association.

[r23] Iversen T. Soskice D. (2001). An asset theory of social policy preferences. American Political Science Review, 95, 875–893.

[r24] Jost J. T. Banaji M. R. (1994). The role of stereotyping in system-justification and the production of false consciousness. British Journal of Social Psychology, 33, 1–27. doi:. 10.1111/j.2044-8309.1994.tb01008.x

[r25] Jost J. T. Glaser J. Kruglanski A. W. Sulloway F. J. (2003). Political conservatism as motivated social cognition. Psychological Bulletin, 129(3), 339–375. doi:. 10.1037/0033-2909.129.3.339 12784934

[r26] Jost J. T. Hunyady O. (2005). Antecedents and consequences of system-justifying ideologies. Current Directions in Psychological Science, 14, 260–265. doi:. 10.1111/j.0963-7214.2005.00377.x

[r27] Jost, J. T., & Kay, A. C. (2010). Social justice: History, theory, and research. In S. T. Fiske, D. Gilbert, & G. Lindzey (Eds.), *Handbook of social psychology* (5th ed., Vol. 2) (pp. 1122-1165). Hoboken, NJ, USA: Wiley.

[r28] Kangas O. (2003). The grasshopper and the ants: Popular opinions of just distribution in Australia and Finland. The Journal of Socio-Economics, 31, 721–743. 10.1016/S1053-5357(02)00143-9

[r29] Korzeniowski, K. (2006). Authoritarianism in Poland in the days of system transformation. In A. Golec de Zavala & K. Skarzynska (Eds.), *Undestanding social change: Political psychology in Poland* (pp. 71-86). New York, NY, USA: Nova Science.

[r30] Kossowska M. Van Hiel A. (2003). The relationship between need for closure and conservative beliefs in Western and Eastern Europe. Political Psychology, 24(3), 501–518. doi:. 10.1111/0162-895X.00338

[r31] Kruglanski, A. (2004). *The psychology of closed mindedness*. New York, NY, USA: Psychology Press.

[r32] Kulin J. Svallfors S. (2013). Class, values, and attitudes towards redistribution: A European comparison. European Sociological Review, 29(2), 155–167. 10.1093/esr/jcr046

[r33] Lepianka D. van Oorschot W. Gelissen J. (2009). Popular explanations of poverty: A critical discussion of empirical research. Journal of Social Policy, 38(3), 421–438. doi:. 10.1017/S0047279409003092

[r34] Lipsmeyer C. S. Nordstrom T. (2003). East versus West: Comparing political attitudes and welfare preferences across European societies. Journal of European Public Policy, 10(3), 339–364. doi:. 10.1080/1350176032000085342

[r35] Mason, D. S. (1995). Justice, socialism, and participation in the postcommunist states. In J. R. Kluegel, D. S. Mason, & B. Wegener (Eds.), *Social justice and political change: Public opinion in capitalist and post-communist states* (pp. 49-80). New York, NY, USA: Walter de Gruyter.

[r36] McFarland S. G. Ageyev V. S. Abalakina-Paap M. A. (1992). Authoritarianism in the former Soviet Union. Journal of Personality and Social Psychology, 63(6), 1004–1010. doi:. 10.1037/0022-3514.63.6.1004

[r37] Örkény A. Székelyi M. (2000). Views on social inequality and the role of state: Posttransformation trends in Eastern and Central Europe. Social Justice Research, 13(2), 199–218. doi:. 10.1023/A:1007502008019

[r38] Redmond, G., Schnepf, S. V., & Suhrcke, M. (2002). *Attitudes to inequality after ten years of transition* (Innocenti Working Paper No. 88). Florence, Italy: UNICEF Innocenti Research Centre.

[r39] Rokeach, M. (1960). *The open and the closed mind*. New York, NY, USA: Basic Books.

[r40] Roosma F. Gelissen J. van Oorschot W. (2013). The multidimensionality of welfare state attitudes: A European cross-national study. Social Indicators Research, 113(1), 235–255. doi:. 10.1007/s11205-012-0099-4 23874057 PMC3696173

[r41] Sabbagh C. Vanhuysse P. (2006). Exploring attitudes towards the welfare state: University students’ views in eight democracies. Journal of Social Policy, 35(4), 607–628. doi:. 10.1017/S0047279406000109

[r42] Sachweh, P., Ullrich, C. G., & Christoph, B. (2007). The moral economy of poverty: On the conditionality of public support for social assistance schemes. In S. Mau & B. Veghte (Eds.), *Social justice, legitimacy and the welfare state* (pp. 123-142). Aldershot, United Kingdom: Ashgate.

[r43] Schaller M. Boyd C. Yohannes J. O’Brien M. (1995). The prejudiced personality revisited: Personal need for structure and formation of erroneous group stereotypes. Journal of Personality and Social Psychology, 68, 544–555. doi:. 10.1037/0022-3514.68.3.544

[r44] Sibley C. G. Duckitt J. (2008). Personality and prejudice: A meta-analysis and theoretical review. Personality and Social Psychology Review, 12(3), 248–279. doi:. 10.1177/1088868308319226 18641385

[r45] Sidanius, J., & Pratto, F. (1999). *Social dominance: An intergroup theory of social hierarchy and oppression.* New York, NY, USA: Cambridge University Press.

[r46] Skocpol, T. (1991). Targeting within universalism: Politically viable politics to combat poverty in the United States. In C. Jencks & P. Peterson (Eds.), *The urban underclass* (pp. 411-436). Washington, DC, USA: The Brookings Institution.

[r47] Suhrcke, M. (2001). *Preferences for inequality: East vs. west* (Innocenti Working Paper No. 89). Florence, Italy: UNICEF Innocenti Research Centre.

[r48] Svallfors S. (1997). Worlds of welfare and attitudes to redistribution: A comparison of eight Western nations. European Sociological Review, 13, 283–304. 10.1093/oxfordjournals.esr.a018219

[r49] Thomassen, J. (2007). Democratic values. In R. J. Dalton & H. D. Klingemann (Eds.), *The Oxford handbook of political behavior* (pp. 418-434). New York, NY, USA: Oxford University Press.

[r50] Todosijević B. (2008). The structure of political attitudes in Hungary and Serbia. Eastern European Politics and Societies, 22(4), 879–900. doi:. 10.1177/0888325408319103

[r51] van Oorschot W. (2000). Who should get what, and why? On deservingness criteria and the conditionality of solidarity among the public. Policy and Politics, 28(1), 33–48. doi:. 10.1332/0305573002500811

[r52] van Oorschot W. (2002). Individual motives for contributing to welfare benefits in the Netherlands. Policy and Politics, 30(1), 31–46. doi:. 10.1332/0305573022501557

[r53] van Oorschot, W. (2008). Popular deservingness perceptions and conditionality of solidarity in Europe. In W. van Oorschot, M. Opielka, & B. Pfau-Effinger (Eds.), *Culture and welfare state* (pp. 268-288). Northampton, United Kingdom: Edward Elgar Publishing.

